# The Japanese version of the Material Values Scale: construct assessment and relationship with age, personality, and subjective well-being

**DOI:** 10.1186/s40359-022-00889-3

**Published:** 2022-08-13

**Authors:** Hiroshi Ohno, Kyung-Tae Lee, Takashi Maeno

**Affiliations:** 1 Graduate School of System Design and Management, Keio University, Kyoseikan, 4-1-1 Hiyoshi, Kohoku-Ku, Yokohama, Kanagawa 223-8521 Japan; 2 Department of Marketing and International Trade, Faculty of Commerce, Chuo University, 742-1 Higashinakano Hachioji-shi, Tokyo, 192-0393 Japan

**Keywords:** Materialism, Material Values Scale, Subjective well-being, Personality traits, Neuroticism, Japan

## Abstract

**Background:**

*Materialism* indicates the extent to which an individual’s life is focused on consumerism, or the acquisition of money and possessions. The Material Values Scale (MVS), comprising the factors “*success,*” “*centrality,*” and “*happiness,*” is a well-known rating scale for materialism. However, a Japanese version of the materialism scale has not yet been established, and the details of the factors and effects related to materialism have not yet been clarified in Japan. The purpose of this study is to develop and evaluate the Japanese version of the MVS (J-MVS).

**Methods:**

We developed the translated J-MVS using a back-translation process. To validate and evaluate the scale based on an online survey, we recruited 500 people, with 100 participants (50 men, 50 women) in five age groups, from 20 to 69 years. We compared and evaluated several factor structure models based on exploratory and confirmatory factor analysis. To evaluate the external criterion-referenced validity of the developed J-MVS scale, we examined the relationship between age, personality, and well-being, which have shown stable relationships with materialism in previous studies.

**Results:**

We developed two six-item dual-factor models. Both models showed significant positive correlations with social comparison orientation and neuroticism, and had significant negative correlations with various subjective well-being indices, suggesting sufficient external criterion-referenced validity. The J-MVS comprising six positive-worded items (J-MVS-P6; without any reverse-worded items) showed a higher correlation with other indicators than the version comprising six items representing all item types and was considered to have higher external criterion-referenced validity.

**Conclusions:**

We propose the J-MVS-P6 for use as a materialism scale in Japan. Compared with the findings from other countries, materialism in Japan may be more closely related to subjective well-being. This scale may be used to examine the effectiveness of various intervention methods for improving individuals’ happiness, based on changes in factors closely related to materialism in Japan.

**Supplementary Information:**

The online version contains supplementary material available at 10.1186/s40359-022-00889-3.

## Background

After World War II, Japan entered a period of rapid economic growth and was considered a prosperous society, with a gross domestic product ranked third globally [1]. Although the country’s economy remains relatively large, its economic growth has slowed recently, with the gross domestic product per capita having dropped to the 25th place globally [1]. This indicates that the nation’s relative economic standing and the global economic environment are changing.

Additionally, Japan was ranked 56th of 104 countries for happiness from 2018 to 2020 [2]. The suicide rates in the country in 2019 were also high compared to other countries, at 15.3 per 100,000 people [3]. Contrarily, the information environment has changed dramatically in recent years as the use of social media has increased worldwide; research shows that the use of social media also increases upward social comparison, which in turn increases materialism [4]. Recent research in Australia has shown that conspicuous consumption increases life satisfaction [5]. Since materialism is a factor suggested to have a close relationship with well-being and that may change along with the information and economic environment, it is necessary to monitor materialism and consider related intervention measures. However, there have been no sufficient observations of materialism in Japan, and it has been difficult to determine how the situation in the country compares with that of other countries; therefore, it is necessary to develop a measurement scale related to this topic.

To understand the relationship between materialism and well-being in Japan, it is also necessary to review the data in the literature and determine whether the characteristics found are regional or are common worldwide. Furthermore, there is the need to identify the factors that influence this relationship to determine what interventions are applicable for supporting people in achieving happiness.

Accordingly, this study developed the Japanese version of the Material Values Scale (J-MVS). To evaluate the external criterion-referenced validity of this scale, we examined the relationship between age, personality, and well-being, which have shown stable relationships with materialism in previous studies.

### Materialism

Materialism is considered a psychological construct related to the emphasis on the acquisition of money, possessions, and status [6, 7]. It has been the focus of attention in the field of consumer research. Belk treated materialism as a personality trait comprising: (1) possessiveness, (2) non-generosity, and (3) envy [6]. However, many subsequent empirical studies have considered materialism as a concept related to values and goals [7, 8]. Kasser’s review of previous research argued that increased materialism not only reduces individual well-being but also increases the likelihood of treating other people and the global environment in ways that undermine the well-being of others; it also describes the possibility of decreasing materialism through interventions to increase people’s happiness [7]. Kasser further proposes that well-being can be changed if more can be learned about materialistic tendencies through empirical evidence clarifying the effects and influencing factors of materialism. Since the details of the influencing factors and effects related to materialism have not been clarified in Japan, this study aimed to develop and evaluate the J-MVS.

### Material Values Scale

Richins and Dawson developed the Material Values Scale (MVS) as a rating scale for materialism in the United States [8]; thereafter, an abbreviated version was developed and evaluated [9]. Exploratory factor analyses showed that the MVS with 18 items and the abbreviated version with 6 to 15 items had a triple-factor structure with moderate goodness-of-fit indices [8, 9]. The three factors were “success,” “centrality,” and “happiness” [8]. The success factor refers to the importance of possession and consumption for success in life, the centrality factor concerns the importance of acquisition and possession, and the happiness factor reflects the belief that possessions and consumption are required for happiness [8]. The internal consistency and external criterion-referenced validity of the MVS and Belk’s materialism scale have been verified and validated [9]. However, the convergent validity analysis showed results that did not entirely meet the average variance extracted (AVE) criterion.

#### Factor structure and reliability of other European versions of the MVS

While Richins and Dawson’s original version of the MVS was shown to have a triple-factor structure in the United States, the same result was not replicated in a study using all 18 items of the MVS in Denmark, France, and Russia [10]. In Denmark, the reduced-item version fit the triple-factor structure and showed sufficient internal consistency; however, the AVEs for each of the domains ranged from 0.37 to 0.51. In both the French and Russian versions, the goodness-of-fit indices of the triple-factor structure were low, and when the items were reduced, the structure that showed better indices was the dual-factor structure (AVEs ranged from 0.30 to 0.47) [10]. In the validation of the scale in German, the goodness-of-fit indices for a dual-factor structure (i.e., success and centrality factors combined and the happiness factor) were higher than that of a triple-factor structure [11]. In this German version, the AVE of the success and centrality factor was estimated to be 0.41, which is below the general criterion based on the reported factor loadings.

#### Factor structure and reliability of Asian versions of the MVS

In Wong et al.’s study [12], using the MVS as a case study, it was shown that when the scales developed in Western countries were translated and used in Asian countries, the responses of participants differed between reverse-worded items (RWI) and positive-worded items (PWI). The correlation between PWI and RWI after reverse coding for RWI was high for American participants, while participants in Asian countries showed weak positive or negative correlations with the translated version of the MVS in an Asian language [12]. It was discussed that the translated Asian mix-worded questionnaire induced participants to respond differently to PWI and RWI, as well as that RWI should be deleted when using Asian-translated scales [12]. A recent study reported a potential acquiescence bias in the PWI-only questionnaire and potentially different understandings of PWI and RWI when they are included in the same questionnaire. Thus, both PWI-only or mixed PWI and RWI questionnaires have disadvantages, entailing that the relationship should be considered a trade-off [13]. A study conducted in Thailand and China attempted to remove RWI and developed a translated version comprising only PWI; this nine-item scale, which had items with low factor loadings removed, was reported to have high goodness-of-fit indices with a triple-factor structure in both Thailand and China [14]. Later, based on research conducted in the United States and Thailand, it was reported that a reduced 10-item, triple-factor, PWI-only version of the scale might be applicable in cross-cultural studies [15]. However, the AVE of the success domain did not meet the convergent validity criterion, having a value of 0.46 in the United States and 0.38 in Thailand. In Japan, although translated and shortened versions of the MVS have been used in some studies (e.g., [16]), the factor structure and reliability of these translated versions have not been verified in detail. In Lee’s study [17], a 9-item shortened Japanese version of the MVS was developed and factor analysis conducted. As a result, a dual-factor structure consisting of a factor for the success and centrality domains and a factor for the happiness domain was developed, as in the German study [11].

Although there are some reports that the translated versions of the MVS in various countries have achieved sufficient internal consistency by deleting items from the original version, there are few reports of the same factor structure with the same 18 items as the original version [10, 15]. In addition, some reports provide insufficient information regarding the validity of the scale, instead focusing on AVE or composite reliability (CR) [10, 14, 15]. The factor structure and response to PWI and RWI may also differ by country due to various factors that have not yet been identified; hence, further verification in multiple countries is necessary.

### Materialism and age

The demographic factor of *age* has been consistently associated with materialism in many previous studies. Materialism has been shown to be higher in younger people [11, 18]. In a meta-analysis of 23 studies, a negative correlation between materialism and age was reported [19]. On the contrary, results of investigating changes in materialism over the life span have reported a curvilinear trajectory that reaches its lowest level in middle age and shows higher levels before and after middle age [19]. It has also been shown that self-uncertainty influences the differences in the degree of materialism by age [20]. In this study, we included the age variable because we examined the external criterion-referenced validity of the developed J-MVS, as well as the relationship of the scale scores with age to clarify the general situation of materialism in Japan.

### Materialism and personality

Regarding the relationship between materialism and personality, it was shown that the Big Five personality traits differed between groups with high and low levels of materialism in American students: the high materialism group was shown to have higher neuroticism and lower extraversion, openness, agreeableness, and conscientiousness [21]. Otero-López et al. reported positive correlations between materialism and neuroticism tendencies, extraversion, and negative correlations with agreeableness and conscientiousness [22]. Subsequently, it was shown that the high materialism group tends to have higher neuroticism and lower agreeableness; the middle group tends to have higher neuroticism and agreeableness or lower neuroticism and agreeableness; the low materialism group tends to have lower neuroticism and higher agreeableness [23]. Furthermore, a relatively strong positive correlation between materialism and social comparison orientation has been reported, especially in comparisons of ability (based on the Iowa Netherlands Comparison Orientation Measure, INCOM) [24].

### Materialism and subjective well-being

Dittmar et al. conducted a meta-analysis focusing on the relationship between materialism and various well-being indicators (e.g., subjective well-being, life satisfaction, self-appraisals, anxiety and depression in DSM, and physical health) [18]. Materialism was negatively correlated with well-being indicator scores, and the strength of its effect on well-being depended on demographic (e.g., sex and age), cultural, and economic factors [18]. It was also suggested that the negative relationship between materialism and well-being was mediated by poor psychological need satisfaction [18]. A longitudinal study conducted with Chinese students by Wang et al. also found a significant negative correlation between materialism and subjective well-being in all three surveys administered [25]. This last cited study also illustrated that materialism indirectly influences subjective well-being and depression through the satisfaction of psychological needs. In sum, the well-being indicators that have been associated with materialism include subjective well-being [26], life satisfaction [18, 25, 26], negative mood [18, 27], stress [26], and physical health [18, 28]. In the current study, several of these indicators were selected and examined.

Whether the relationships between materialism, personality, and various well-being indicators are valid in Japan has not yet been fully clarified. We predict that materialism will be negatively correlated with the scores of various well-being indicators and closely related to personality, especially to social comparison orientation and neuroticism in Japan. To evaluate the external criterion-referenced validity of the J-MVS, it is necessary to investigate whether these predicted tendencies are replicated.

### Aims and research questions

This study aimed to develop and evaluate the J-MVS and assess its external criterion-referenced validity. The research questions are as follows:

- Does the J-MVS exhibit the same triple-factor structure as the original version of the MVS? If not, what factor structure does it exhibit?
- Does the J-MVS have enough internal consistency, validity, and reliability?
- How does materialism measured by the J-MVS relate to age and personality? Is materialism inversely related to age, as in previous studies? Does materialism tend to be higher in those with high neuroticism and lower in those with high agreeableness? Does social comparison orientation correlate as highly as predicted?
- Does materialism, as measured by scores for the J-MVS, correlate negatively with subjective well-being indicators scores, as in previous studies? How does the extent of each of these correlations compare with previous reports from other countries?

## Methods

### Ethics

Participants were guaranteed anonymity by the online survey agency responsible for sample recruitment, and we did not handle personally identifiable information such as names. Participation in this study was voluntary, and only those who agreed to the possibility that their individual data might be published in an anonymous survey were asked to complete the questionnaire. This study was approved by the ethics committee of the Graduate School of System Design and Management, Keio University.

### Participants and procedures

In 2019, participants were recruited from among the 320,000 registered members of a Japanese online survey agency that offered reward points. In this case, the reward points were equivalent to 70 yen (0.55 US dollars as of May 2022). The points could be exchanged for Amazon gift certificates, shopping coupons, and more. The agency asked participants to complete a web-based questionnaire called “Research on perceptions of yourself and society” and select from response options they most agreed with. Based on research findings on response quality, to improve the quality of the responses, participants were requested to agree to read the questions carefully and answer them conscientiously [29]. We also included two dummy questions (attention check items: e.g., “please select ‘10’ for this question”) for this purpose.

The survey was terminated when the number of participants reached 500 in total, such that there were equal numbers of men and women in each age group after excluding those who did not agree to participate or those who gave inappropriate responses for dummy questions. The 500 comprised 100 (50 men, 50 women) in each age group from 20 to 69 years (i.e., 20–29, 30–39, etc., through 60–69). For data analysis, 500 was the goal number for participants because generally, assuming an infinite population, a sample size of about 400 is required to obtain data with a confidence coefficient of 95% and an allowable error of 5%; therefore, data from 500 individuals were collected to ensure data would come from more than 400 participants.

In determining the 500-person data set, 168 of the initial respondents were eliminated by the online survey agency due to the following reasons: 150 had provided wrong answers to the dummy questions, seven had consistently and consecutively selected the same choice in more than three question groups (one question group means one scale group), and 11 were randomly chosen for removal based on the contract for delivering data from 500 participants to the researchers.

Participants were required to provide demographic information such as sex, age, area of residence, marital status, employment status, employment type, education level, number of family members living together, annual household income, and number of friends (Additional file 1: Table 1). The median value of each option was used in the numerical data processing of annual household income and number of friends. We used the value estimated by Parker and Fenwick’s estimation formula for the open-ended option for income [30]. For open-ended responses regarding the number of friends (i.e., > 31), we treated the number as 35 for convenience and so we could control for extreme results. The items included in the questionnaires are described below.

**Table 1:** Factor loadings for the J-MVS-A6 and J-MVS-P6 (N = 500)

			Factor loadings for the J-MVS-A6		Factor loadings for the J-MVS-P6	
Item num.	Domain	Item	Success/Centrality	Happiness	Success/Centrality	Happiness
1	S/C	I admire people who own expensive homes, cars, and clothes. (3, 6, 9, 15)	0.75		0.74	
5	S/C	I like to own things that impress people. (9, 15)	–		0.49	
12	S/C	I like a lot of luxury in my life. (3, 6, 9, 15)	0.71		0.72	
15	H	My life would be better if I owned certain things I don’t have. (6, 9, 15)		0.75		0.72
16	H	I wouldn’t be any happier if I owned nicer things. (15) R		0.55		–
17	H	I’d be happier if I could afford to buy more things. (3, 6, 9, 15)		0.88		0.88
18	H	It sometimes bothers me quite a bit that I can’t afford to buy all the things I’d like. (9, 15)		0.65		0.67

		Phi matrix: happiness	0.55		0.55	
		Mean	2.44	3.20	2.42	3.18
		SD	1.01	0.84	0.89	0.92
		Skewness	0.31	0.22	0.28	0.14
		Kurtosis (difference from 3.00)	− 0.65	− 0.23	− 0.49	− 0.39
		Mean (overall)	2.95		2.80	
		SD (overall)	0.76		0.77	
		Skewness (overall)	0.27		0.20	
		Kurtosis (difference from 3.00) (overall)	− 0.25		− 0.24	

Numbers in parentheses indicate items adopted in various abbreviated versions of the original version (e.g., “3” means the 3-items version of MVS) [9]

R, reversed items

### Measures

#### Materialism

We adopted the following process for the translation of the J-MVS, which involved three collaborators [8, 9]. First, the Japanese translation was prepared with the cooperation of the first collaborator, who is fluent in both Japanese and English. Second, the Japanese translation was back-translated into English by the second collaborator, also skilled in Japanese and English. Finally, a native English speaker verified the back-translation; we received comments from him and made corrections to the Japanese translation, resulting in the J-MVS. As in the original MVS, we adopted a five-point Likert response scale with higher scores indicating higher materialism. The J-MVS is shown in Additional file 2.

#### Personality traits

##### Big five personality

We used the Japanese version of the Ten Item Personality Inventory (TIPI-J) [31, 32], which measures the Big Five personality traits with two items each. Participants were asked to rate items related to extraversion, agreeableness, conscientiousness, openness, and neuroticism (reversed scores of the emotional stability item in the TIPI) using a 7-point scale (1 = disagree completely; 7 = strongly agree). For reversed items, the scores were reversed. Each personality factor was calculated on a 14-point scale. The higher the total score, the stronger the personality tendency corresponding to each factor.

##### Social comparison orientation

The Japanese version of Gibbons and Buunk’s INCOM was used to assess social comparison orientation, and participants were required to answer a total of 11 items using a 5-point scale (1 = disagree completely; 5 = strongly agree) [33, 34]. In the Japanese INCOM, unlike the original version, item 11 measures ability comparison, and item 9 measures both ability comparison and opinion comparison. Therefore, item 9 was excluded from the scale scores, and the scores of seven items for ability comparison and three items for opinion comparison were calculated. A higher INCOM score indicates a higher social comparison orientation.

#### Subjective well-being

##### Self-rated happiness

We used the question used by the Cabinet Office in Japan [35]: “How happy are you with yourself at present?” To improve the sensitivity of responses, we adopted an 11-point scale (0 = very unhappy; 10 = very happy). For the purposes of this study, we refer to this item as measures of “self-rated happiness.” We also used the Japanese version of Lyubomirsky et al.’s Subjective Happiness Scale (SHS) [36, 37], consisting of four items. Higher SHS scores indicate higher levels of happiness.

##### Life satisfaction

The Japanese translation of the Satisfaction with Life Scale (SWLS) by Diener et al. was used as an indicator of satisfaction with life [38, 39]. A total of five questions were used as the SWLS score, with higher SWLS scores indicating higher levels of satisfaction.

##### Meaning in life

The Meaning in Life Questionnaire (MLQ) by Steger et al. is used as an index to measure the sense of meaning in life [40]. The MLQ consists of five items corresponding to the subscale of Presence of Meaning in Life (MLQ Presence), which measures the subjective sense that one’s life is meaningful, and five items corresponding to the subscale of Search for Meaning in Life (MLQ Search), which measures the drive and orientation toward finding meaning in one’s life [40]. The statistical properties of the Japanese version of the MLQ were clarified by Steger et al. [41]. The Japanese version used by Steger et al. that we employed in this study was provided by Shimai, who was part of Steger’s research group [41]. The items are responded on a 7-point scale; the total scores of the answers to the five questions in each sub-scale were used as the MLQ Presence score and the MLQ Search score.

##### Negative affect

The negative affect section of the Japanese version of the Positive and Negative Affect Schedule (PANAS) was used as a scale for self-assessment of current negative mood [42]. The order of the items was randomized in accordance with the guidelines for its use. The response scale ranged from 1 to 6 (1 = completely disagree; 6 = strongly agree).

##### General health

Regarding self-assessment of general health, we asked participants: “How do you currently feel about your health?” The item was answered on an 11-point scale ranging from 0 to 10 (0 = not healthy at all; 10 = very healthy) [35]. For the purposes of this paper, we refer to this as “self-rated health.”

##### Stress

As a measure of self-assessed stress, we presented participants with a question based on Watson’s brief index [43]: “How much stress (e.g., because of hassles and demands) were you under recently?” To improve the sensitivity of responses, we asked the participants to answer to this item on an 11-point scale, ranging from 0 to 10 (0 = didn’t feel stress at all; 10 = felt stress very much). For the purposes of this study, we refer to this as “self-rated stress.”

##### Others

The questionnaires also included items on ideal happiness [44–47], subjective socioeconomic status [48], the Japanese version of the Relative Deprivation Scale [49], the Scale for Independent and Interdependent Construal of Self [50], and the Japanese version of the Sense of Social Support Scale [51]. However, they are not included in the content reported in this paper; this is because our focus is on the structural evaluation of the developed scale and its external criterion-referenced validity, which were conducted based on analyses of the correlation of the scale score with the scores for personality and various well-being indicators.

### Analyses

For main statistical processing, R software, version 4.0.1, was used. The Psych package was used for parallel analysis and exploratory factor analysis, the Lavaan package was used for confirmatory factor analysis, and the semTools package was used to calculate reliability coefficients (Cronbach’s α, CR, and AVE). Criteria of goodness-of-fit indices of models were based on Hooper et al.’s report [52], with root mean square error of approximation (RMSEA) < 0.08, adjusted goodness of fit index (AGFI) ≥ 0.90, standardized root mean squared residual (SRMR) ≤ 0.08, Tucker-Lewis Index (TLI) ≥ 0.95, and comparative fit index (CFI) ≥ 0.95. For CR, which represents the scale’s internal consistency, a value of > 0.7 was used as the criterion, as indicated by Hair et al. [53]. For AVE, which represents the scale’s convergent validity, a value of ≥ 0.5 was used as the criterion, as indicated by Bagozzi and Yi [54]. Measurement invariance tests were conducted using AMOS 28.

## Results

### Descriptive statistics for items in the J-MVS

Descriptive statistics for all 18 items included in the J-MVS are shown in Additional file 1: Table 2. Only in the absolute value of item C1 (item 1 of the centrality domain) did the skewness exceed 0.5, indicating that it was slightly skewed. The skewness of all other items, particularly those in the happiness domain, was close to 0. The mean scores for each questionnaire item ranged from 2.17 to 3.55, and the standard deviations ranged from 0.88 to 1.22.

**Table 2:** Correlation between the J-MVS-A6 or J-MVS-P6 and subjective well-being measures (N = 500)

			Self-rated general health (11-point scale)	Self-rated stress (11-point scale)	Negative affect (PANAS)	Self-rated happiness (11-point scale)	Subjective happiness scale (SHS)	Life satisfaction (SWLS)	Presence of meaning in life (MLQ-presence)	Search for meaning in life (MLQ-search)
		Mean	5.94	6.14	22.46	5.99	4.37	18.37	18.78	21.75
		SD	2.44	2.49	9.21	2.37	1.19	6.58	6.74	6.14
		α	–	–	0.93	–	0.84	0.90	0.90	0.90
J-MVS-A6	Overall	Pearson's r	− 0.03	0.26***	0.28***	− 0.31***	− 0.36***	− 0.36***	− 0.17**	0.16**
	Success/centrality		− 0.02	0.12	0.21***	− 0.10	− 0.13	− 0.11	0.01	0.22***
	Happiness		− 0.03	0.28***	0.26***	− 0.37***	− 0.41***	− 0.42***	− 0.24***	0.09
J-MVS-P6	Overall	Pearson’s r	− 0.03	0.23***	0.33***	− 0.29***	− 0.34***	− 0.30***	− 0.11	0.23***
	Success/centrality		0.00	0.11	0.25***	− 0.10	− 0.16*	− 0.08	0.06	0.30***
	Happiness		− 0.05	0.28***	0.30***	− 0.39***	− 0.42***	− 0.42***	− 0.24***	0.09

Evaluation by adjusted *p* value based on Holm method

***p < .001, **p < .01, *p < .05

As in the original version, the 18-item and 15-item versions are tentatively referred to as the J-MVS-18 and J-MVS-15. Factor loadings, reliability and validity indices, and model fit indices based on confirmatory factor analysis are shown in Additional file 1: Table 2. In both the J-MVS-18 and J-MVS-15, CRs in the centrality domain were 0.60 and 0.52 and AVEs were 0.20–0.45, which did not meet the criteria. In addition, the goodness-fit-indices were low, and most did not meet the criteria.

### Confirmatory factor analysis of the J-MVS and evaluation of reliability

Since the results of confirmatory factor analysis for the J-MVS-18 and J-MVS-15, which are translated versions of the original versions, were not good (as described in the previous section), exploratory factor analyses were conducted to identify the factor structure. Exploratory factor analyses were conducted on the full set of 18 items as in the original version, or on a 10-item set consisting of only the PWI; we chose to conduct the analyses based solely on PWI based on the report that RWI show different characteristics, especially in Asian countries [12]. Exploratory factor analyses were conducted repeatedly by setting the following criteria and deleting items that did not meet the criteria until convergences were achieved with only items that met the criteria. We attempted to extract factors by setting the cutoff criterion for factor loadings to 0.3, 0.4, or 0.5, and the cutoff criterion for communality to 0.2 or no cutoff criterion for communality for each. After converging on only the items that met the criteria, confirmatory factor analyses were conducted. Additional file 1: Table 3a shows the results of confirmatory factor analyses for the factor structure obtained by exploratory factor analysis with each cutoff criterion for the 18 items (models 1–6), and Additional file 1: Table 3b shows the results for the 10 PWI items (models 7–12). Models 3 and 4 were identical as a result of these analyses.

As a result of confirmatory factor analyses, model 5 (18 items) and model 11 (10 PWI items) met the criteria for the goodness-of-fit indices, excluding models 10 and 12, which extracted only the happiness domain. In both models (model 5 and 11), two factors were extracted: the success/centrality factor—which had higher loadings in the success domain items or the centrality domain items—and the happiness factor—which had higher loading in the happiness domain.

Model 5 consisted of questions 1, 12 (success/centrality domain) and 15, 16, 17, 18 (happiness domain). The AVEs of the two domains were 0.518 and 0.538, meeting the criterion, and the CRs were 0.698 and 0.805, which were slightly below the criterion for the success/centrality domain. The alpha coefficient was 0.787, showing sufficient internal consistency.

Model 11 consisted of questions 1, 5, 12 (success/centrality domain) and 15, 17, 18 (happiness domain). The AVEs of both domains were 0.448 and 0.575, slightly below the criterion in the success/centrality domain, and the CRs were 0.700 and 0.809, meeting the criterion. One of the criteria for discriminant validity is that the AVE should be greater than the square of the correlation coefficient between each factor (Fornell and Larcker criterion) [55]; since the inter-factor correlations were 0.55 and its square was 0.30 in both models 5 and 11, the AVEs were greater than the square, so they met the criterion. The alpha coefficient was 0.776, showing sufficient internal consistency.

The goodness-of-fit indices of the two dual-factor structure models (models 5 and 11) validated by confirmatory factor analysis were better than those of other models. The indices of internal consistency and validity of the domains were sufficient or better than those of previous reports, although some of them were slightly below the criteria. Therefore, the J-MVS was considered to have a dual-factor structure; hereinafter, models 5 and 11 will be referred to as J-MVS-A6 and J-MVS-P6, respectively, and will be examined for external criterion-referenced validities. The factor loadings and descriptive statistics for each question in the J-MVS-A6 and J-MVS-P6 are shown in Table 1. The absolute values of skewness and kurtosis (difference from 3) of the scale scores of the six items in both models were less than 0.5, indicating a normal distribution.

### Relationship between the J-MVS, age, and personality

We conducted correlation analysis for the relationship between J-MVS scores and age, the INCOM scores, and TIPI-J scores. Results showed lower materialism with increasing age and higher materialism with higher social comparison orientation and neuroticism. The correlation coefficients between the J-MVS-A6 and J-MVS-P6 scores, the success/centrality domain and happiness domain scores, age, INCOM scores, and TIPI-J scores are shown in Additional file 1: Table 5. The alpha coefficients of the TIPI-J, which consists of two items for each personality trait, were low and ranged from 0.34 to 0.60.

Significant negative correlations were found between J-MVS-A6 and J-MVS-P6 scores and age in the overall scale and the domains; however, the correlation coefficients in the success/centrality domain were relatively weak. Significant positive correlations of both J-MVS-A6 and J-MVS-P6 scores with the ability comparison scores were confirmed in the overall scale and its domains. Regarding opinion comparison scores, positive correlations with the overall scale and the success/centrality domain scores were found in the J-MVS-A6 and J-MVS-P6.

Significant positive correlations were found with neuroticism, one of the Big Five personality traits, in both the overall scale and the happiness domain scores; weak positive correlations were found in the success/centrality domain. As for agreeableness, weak negative correlations with the overall scores and happiness domain scores of the J-MVS-A6 and J-MVS-P6 were found. We found a positive correlation between openness and the J-MVS-P6 success/centrality domain score; we also confirmed a weak negative correlation between conscientiousness and the J-MVS-P6 happiness domain score.

Comparing the J-MVS-A6 and J-MVS-P6, although the differences were small, the latter had higher correlations with ability comparison (r = 0.51 in the overall scale), opinion comparison (r = 0.29 in the success and centrality domain), and neuroticism (r = 0.28 in the happiness domain) than the former. The J-MVS-A6 showed a higher correlation with agreeableness (r = − 0.17 in the overall scale); however, the difference between this result and the correlation with the J-MVS-P6 was only of 0.02.

### Relationship between the J-MVS and subjective well-being

The relationship between J-MVS-A6 and J-MVS-P6 scores and various subjective well-being indices was examined by correlation analysis. We found that the higher the materialism measured by the J-MVS, the lower the subjective well-being.

The correlation coefficients between the scores for the J-MVS-A6 and J-MVS-P6, including the success/centrality and happiness domains, and various subjective well-being indices—self-rated happiness (single scale), SHS, SWLS, MLQ scale, PANAS, self-rated general health, and self-rated stress scales—are shown in Table 2. The alpha coefficients for the various well-being indices were sufficiently high, ranging from 0.84 to 0.93.

The J-MVS-A6 and J-MVS-P6 and their domain scores did not correlate with self-rated health but were significantly and negatively correlated with self-rated happiness, SHS, and SWLS scores. On the contrary, self-rated stress levels and PANAS scores showed significant positive correlations with the J-MVS-A6 and J-MVS-P6. Additionally, there were significant negative correlations between overall scores, happiness domain scores, and MLQ Presence, and significant positive correlations between overall scores, success/centrality domain scores, and MLQ Search.

Comparing the J-MVS-A6 with the J-MVS-P6, although the differences were small, the latter scores showed higher correlations with scores for self-rated happiness (r = − 0.39 in the happiness domain), the SHS (r = − 0.42 in the happiness domain), the PANAS (r = 0.33 in the overall scale), and the MLQ-Search (r = 0.30 in the success/centrality domain) than the former.

### Measurement invariance test of the J-MVS-P6

As described in detail in the Discussion section, we judged that the J-MVS-P6 was superior to the J-MVS-A6 in terms of external criterion referenced-validity and item structure. Hence, we conducted the measurement invariance test for the J-MVS-P6 (Additional file 1: Table 7) to check whether the obtained factorial structure would be stable in different sex and age groups (20 s, 30 s, 40 s, 50 s, and 60 s). For sex groups, there was no significant worsening of the χ square, CFI, and RMSEA not only between the configural invariance model and the partial metric invariance model (Δχ^2^ = 4.740; ΔCFI = 0.000; ΔRMSEA = − 0.003), but also between the partial metric invariance model and the partial scalar invariance model (Δχ2 = 5.81; ΔCFI = − 0.003; ΔRMSEA = − 0.001). For age groups, there was no significant worsening of the χ square value, CFI, and RMSEA between the configural invariance model and the partial metric invariance model (Δχ2 = 21.364; ΔCFI = − 0.001; ΔRMSEA = − 0.005).

## Discussion

In this study, we conducted an initial evaluation of the J-MVS, a new Japanese version of the MVS that we developed with data from 500 people in Japan, while controlling for age and sex. No materialism scale has been examined for use in Japan in any previous research. The following is a discussion of each of the research questions proposed at the beginning of this paper.

The J-MVS was considered to have a dual-factor structure based on the following points: (1) the triple-factor structure, which was the same as the original 18-item version and the subsequently proposed 15-item version, had a low goodness-of-fit; (2) the J-MVS-A6 and J-MVS-P6 were the most dominant among the models compared in this study. The content of those versions was derived through exploratory factor analyses, and they were shown to meet the various goodness-of-fit indices.

Both the J-MVS-A6 and J-MVS-P6 were shown to have sufficient internal consistency. The discriminant validity of the success/centrality domain and the happiness domain was also demonstrated. The convergent validity of the J-MVS-P6 was slightly below the criterion but was considered to be relatively good when referring to previous reports from other countries. Based on the factor loadings reported in the German version [11], the AVE in the success/centrality domain was estimated to be 0.41, and, in Watchravesringkan’s validation in the United States and Thailand, the AVE in the success domain was less than 0.50 [15]. The AVE calculated from the factor loadings based on Richins’ original 18-item [8] exploratory factor analysis was 0.36–0.41 for each domain, whereas the J-MVS-P6 showed an AVE of 0.45.

Regarding CR, the J-MVS-P6 met the criterion, while the J-MVS-A6 had CR close to the criteria (0.698 in the success/centrality domain). In both these scales, the goodness-of-fit indices of the dual-factor structure were high, and although some of the validity and reliability indices did not fully meet the criterion mentioned above, they were considered acceptable at this stage when compared to existing reports. Nevertheless, the fact that there are many items with low factor loadings and insufficient validity indices in translated versions of the MVS is an issue, not only for the developed J-MVS but also for other language versions of the MVS in general.

The reasons for the J-MVS being judged as having a dual-factor structure, not a triple-factor structure as in the original version, require further investigation. Griffin et al. suggested that the triple-factor structure was not replicated in France and Russia for the MVS because the inter-factor correlations were high, and there may have been a lack of discrimination between the factors [10]. They also suggested that, according to previous studies, RWI can cause problems in cross-cultural studies, potentially leading some items to not have sufficient factor loadings [10]. In this study, the above situation may have contributed to the dual-factor structure. Furthermore, the division of materialism into two factors may be consistent with the ideas described in the “Dual Model of Materialism” [56, 57] by Sirgy et al. In this model, the two aspects of materialism, success materialism and happiness materialism, are clearly distinguished. Success materialism is related to economic motives and positively influences life satisfaction, while happiness materialism may increase dissatisfaction with the current standard of living [56, 57].

When scales developed in Western countries are translated, the responses of individuals in Asia differ between PWI and RWI, and one of the proposed solutions for this conundrum was the deletion of RWI [12]. Hence, to construct a more appropriate scale and verify the external criterion-referenced validity, we used both the J-MVS-A6, which was obtained by repeating the exploratory factor analysis for the original 18 items, and the J-MVS-P6, which was obtained by repeating the exploratory factor analysis for the items without the RWI.

When the J-MVS-P6 was compared with the J-MVS-A6, the correlation level with personality (social comparison orientation, neuroticism) and subjective well-being indices were almost the same or slightly higher in the J-MVS-P6. Therefore, the external criterion-referenced validity of the J-MVS-P6 was the same or slightly higher than that of the J-MVS-A6. In addition, Wong et al. discussed that the questionnaire composed only of PWI is less likely to confuse respondents [12]. Furthermore, in the J-MVS-P6, the number of measurement items is well balanced, with three items in each domain. We therefore propose the J-MVS-P6, which consists of six items (three items from the success/centrality domain and three items from the happiness domain) and has no RWI, as a scale to measure materialism in Japan.

The distribution of scores was close to a normal distribution, making the scale easy to use in research. However, it should be noted that there are some disadvantages of using PWI alone, such as the increase in straight-line responses and the possibility of agreement bias [12, 13]. The results of the measurement invariance test supported configural invariance, partial metric invariance (i.e., the equivalence of factor loadings), and partial scalar invariance (i.e., the equivalence of intercepts between sex groups). Regarding age groups, although the measurement invariance test was conducted between relatively small-sized sample groups (100 each), it was supported up to a partial metric invariance, indicating the equivalence of factor loadings. This is considered to confirm a degree of measurement invariance.

Regarding the relationship between the MVS and age, a meta-analysis of 23 existing studies reported a mean correlation coefficient of − 0.16 with a 95% confidence interval of − 0.14 to − 0.18 [19]. The correlation coefficient between the J-MVS-P6 and age was − 0.28, indicating a higher correlation than that in prior research. Regarding the association between the J-MVS-P6 and ability comparison in the INCOM, the association was high with r = 0.51; this number is close to the value reported by Kim et al. (r = 0.60) [24].

Regarding the correlation between the MVS and neuroticism (one the Big Five personality traits), it was previously reported to be r = 0.22–0.24 for the overall scale [23], r = 0.33 for the overall scale, r = 0.36 for the happiness domain, and r = 0.12 for the centrality domain [58]. In the present study, positive correlations with neuroticism were found in the J-MVS-P6 overall score (r = 0.27), the happiness domain score (r = 0.23), and the success/centrality domain score (r = 0.14). As for the correlations of the MVS with agreeableness and conscientiousness, negative correlations were suggested in previous studies [21, 22]. In this study, the J-MVS-P6 overall scores and the happiness domain scores were also negatively correlated with agreeableness scores; the J-MVS-P6 happiness domain score was also negatively correlated with the conscientiousness score. Many of the previously reported tendencies in the relationship between materialism, age, and personality were replicated. Negative, weak positive, and insignificant correlation coefficients have been reported for the MVS with agreeableness, extraversion, and openness, respectively [22]. In the J-MVS-P6, no significant correlations were found with agreeableness and extraversion, and a weak positive correlation with openness was found in the success and centrality domain. It has been reported that low materialism groups have higher openness [21], and based on regression analysis, it has also been shown that low openness predicts high materialism [22]. Therefore, the positive correlation with openness in the success and centrality domains of the J-MVS-P6 may be a Japanese-specific tendency. Kilbourne et al. hypothesized that openness is positively related to variety and excitement associated with consumption, which leads to higher materialism [59]. The positive association between openness and materialism found in the present study may be consistent with the mechanism hypothesized by Kilbourne et al. [59].

The J-MVS-P6 scores were negatively correlated with various well-being indicator scores (except for self-rated health), and the negative correlation was particularly large in the happiness domain. The mean correlation coefficient between materialism and well-being was reported as − 0.15 in a meta-analysis by Dittmar et al. [18]. The correlation coefficients between the J-MVS-P6 overall score and self-rated happiness, SHS, and SWLS scores ranged from − 0.29 to − 0.34; those with the happiness domain ranged from − 0.39 to − 0.42, which were larger than those reported in the meta-analysis [18]. These results suggest that materialism and well-being may be more closely related in Japan.

The correlation between the J-MVS-P6 overall score and PANAS score was r = 0.33, which was comparable to that in a previous report (r = 0.35) [27]. For the correlation with self-rated stress, the r = 0.23, which was similar to the significant correlation coefficient of 0.20 with the MVS score reported in Burroughs and Rindfleisch’s study [26]. However, and in contrast to the results found in Western countries and Korea [18, 28], the J-MVS-P6 score was shown to be nearly uncorrelated with self-rated general health.

The J-MVS-P6 and the MLQ Search scores were positively correlated, and a higher positive correlation was found with scores for the success/centrality domain. It may be that the tendency to search for meaning in life may be higher among people who consider material wealth to be a success and a central value in life. The higher the tendency to regard material possessions and consumption as life success or as a central value in life, the more likely it is to lead to dissatisfaction with psychological needs [25]; this may increase the tendency to search for meaning in life.

In contrast to the results from a study conducted with American people, the values for the MLQ Search and MLQ Presence were positively correlated (r = 0.24) among Japanese people [41]. In the present study, both were highly and positively correlated (r = 0.44); however, the J-MVS-P6 score showed different associations with MLQ Search and MLQ Presence scores. Specifically, the happiness domain and MLQ Presence scores showed a negative correlation. These results suggest that people with high materialism tend to have a weaker sense of presence of meaning in their lives. Further, the higher the tendency to think of material possession and consumption as happiness, the more likely it is that the presence of meaning in life is weakened due to dissatisfaction with psychological needs. The evidence on the relationship between the MLQ Presence and the MLQ Search differs between the United States, which has a high independent construal of self, and Japan, which has a high interdependent construal of self [41]. The different tendencies between the MLQ Presence and the MLQ Search scores in the success/centrality domain and the happiness domain of materialism may be based on differences in the cultural construal of self.

### Limitations

To ensure the equivalence of the translated scale with the original scale, we obtained the cooperation of three people and performed a back-translation process. However, there may have been a tendency to be overly literal in the interpretations. This tendency may be related to the low factor loadings on some of the scale items. For future scale translations, the challenge will be to make the wording more natural and easier to understand while ensuring equivalence, for example, by utilizing more human resources or AI.

Additionally, respondents were limited to those who responded voluntarily to the online agency’s call for responses, use the Internet in their daily lives, and are registered as survey company monitors. At the same time, the weakness of the argument regarding sample size could be pointed out, and it might have been better to determine the sample size using a power analysis approach. Therefore, the results may be limited in their generalizability for the Japanese population. The situation of those who are not proficient or comfortable with computers or other online (internet) interfaces and those who do not intend to register as a survey company’s monitor will be important data and should be investigated in the future. It will be necessary to conduct interviews or paper-based surveys to analyze subjects according to their demographics.

In addition, to verify the external referenced-criterion validity of the scale, many indicators were incorporated into the questionnaire, but the large number of questions may have reduced the quality of the responses. In the future, it would be desirable to conduct a survey with the number of questions below a certain level.

Furthermore, while sufficient reliability was confirmed for the well-being and social comparison orientation measures, the reliability of the Big Five scale with two items for each personality trait was low. This also affects the reliability of the correlation with the J-MVS-P6. Future researchers will have to tackle the challenge of using a more reliable version of the Big Five questionnaire instead of a shortened version, such as the TIPI-J.

## Conclusion

This study aimed to translate Richins and Dawson’s MVS to develop the J-MVS and verify the validity of the scale’s structure and external referenced-criterion validity [8]. The J-MVS-P6 (The J-MVS comprising six PWI) developed in this study did not have sufficient goodness-of-fit-indices when all 18 items from the original version were included, and the triple-factor structure was not confirmed. In contrast, the suitability of the triple-factor structure was confirmed by Richins in their study for use of the scale in the United States [8]. However, the triple-factor structure has not been replicated in subsequent studies, including the German version and the present study [11]. Future research should further examine whether the extraction of centrality and success factors separately is a phenomenon specific to some regions, such as the United States, and whether there are common global and region-specific factors that constitute materialism.

In addition, in Thailand [15], Germany [11], Denmark, and other countries [10], the AVE of the MVS they developed did not meet the criteria and some items with low factor loadings were found. In the present study, factor loadings of some items were also low, and as a result of deleting items, only six items were found to be valid for use in the scale. It seems difficult to ensure that the materialism scales developed and studied in various countries, including the current scale, have sufficient equivalence with the original version. Therefore, there is a need for a scale that is sufficiently equivalent and comparable for use in various cultures. Since the reliability of the J-MVS-P6 was limited due to the small number of items, it may be necessary to construct a more reliable set of items and evaluate it in more countries. In the present study, as proposed by Wong et al. [12], the strength of external criterion-referenced validity seemed to increase slightly by deleting the RWI; hence, it is necessary to consider a scale structure in which RWI are deleted.

Nevertheless, there were clear negative correlations between scores for the J-MVS-P6—including subscales scores—and subjective well-being indicators among participants in Japan. Hence, currently, materialism may be more closely related to subjective well-being in Japanese culture than in other cultures. The relationship between J-MVS-P6 scores and personality, such as neuroticism and social comparison orientation, was similar to the relationship described in previous reports. These results suggest that the J-MVS-P6 has enough external referenced-criterion validity.

The J-MVS-P6 showed relatively good internal consistency and CR after deleting RWI and low-factor loading items. Therefore, we propose the J-MVS-P6 as a tentative measure of materialism in Japan. Using the J-MVS-P6, which has confirmed structural compatibility and external criterion-referenced validity, it will be possible to monitor how the materialism of individuals and groups in Japan changes as the information and economic environments change. It can also be used to examine the relationship between these changes and the health and happiness of Japanese citizens.

As for the relationship between the J-MVS-P6 and other factors, the present study was limited to correlation analysis, and we see the need to compare our findings for these relationships with various other analyses conducted in other countries. It has been identified that materialism is associated with psychiatric symptoms such as compulsive buying [11], depression, and anxiety [7]; hence, future research should clarify how materialism is related to these psychiatric symptoms in Japan. It has been suggested that materialism has a greater impact on well-being in Japan; an investigation into how materialism fluctuates in the long term due to various factors, such as the social and media environments in Japan, will be required in the future.

This study was the first in Japan to translate and validate all items of the MVS and propose a set of scales that can be used in future research. The scale may be used to examine the effectiveness of various intervention methods for improving happiness based on changes in factors closely related to materialism for individuals in Japan.

## Supplementary Information

**Additional file 1.** This file contains tables too wide for an A4/Letter page.

**Additional file 2.** The Japanese version of the Material Value Scale (J-MVS). This file contains all MVS items translated into Japanese.

## Data Availability

The data set supporting the conclusions of this article is available on reasonable request from the corresponding author. For all Japanese scales in our study other than those for which permission is not required, we have received permission to use them from the developers. Although we would like to present the Japanese versions of all the scales used as additional materials, in Japan, all researchers who developed the Japanese version of the scale need to provide their further permission to reprint them. Therefore, although we formally cited the literature, we have refrained from presenting the scales for reprinting. For the original English version of MVS, we have obtained permission from the developer to describe the Japanese translation of MVS in conjunction with the English version.

## Abbreviations

MVS: The Material Values Scale
J-MVS: The Japanese version of MVS
J-MVS-P6: The J-MVS comprising six positive worded items
AVE: Average variance extracted
PWI: Positive-worded items
RWI: Reverse-worded items
CR: Composite reliability
INCOM: Iowa Netherlands Comparison Orientation Measure
TIPI-J: The ten item personality inventory
SHS: The Subjective Happiness Scale
SWLS: The Satisfaction with Life Scale
MLQ: The Meaning in Life Questionnaire
MLQ Presence: Presence of meaning in life
MLQ Search: Search for meaning in life
PANAS: The positive and negative affect schedule
RMSEA: Root mean square error of approximation
AGFI: Adjusted Goodness of Fit Index
SRMR: Standardized root mean squared residual
TLI: Tucker-Lewis Index
CFI: Comparative Fit Index
J-MVS-A6: The J-MVS comprising six positive and reversed items
