# Supplementary material for: The Japanese version of the Material Values Scale: construct assessment and relationship with age, personality, and subjective well-being
Source: BMC Psychol. 2022 Aug 13;10:200. doi: 10.1186/s40359-022-00889-3 (PMC9375416; doi:10.1186/s40359-022-00889-3)
Supplement: Supplementary file 1 — Additional file 1. This file contains tables too wide for an A4/Letter page. [file 40359_2022_889_MOESM1_ESM.docx]

Table 1. Sample characteristics

| *N* | 500 |  | Working (%) |  |
| --- | --- | --- | --- | --- |
| *M* age (*SD*) | 44.83 (14.105) |  | Working | 70.6 |
| Number of family members living together (%) | |  | Not working | 29.4 |
| Living alone | 15.2 |  | Income (%) |  |
| Living with 1 | 35.4 |  | < 2,000,000JPY | 9.4 |
| Living with 2 | 24.0 |  | ≥ 2,000,000JPY; < 3,000,000JPY | 8.2 |
| Living with 3 | 16.6 |  | ≥ 3,000,000JPY; < 4,000,000JPY | 13.4 |
| Living with 4 | 6.8 |  | ≥ 4,000,000JPY; < 6,000,000JPY | 17.2 |
| Living with 5 | 1.2 |  | ≥ 6,000,000JPY; < 8,000,000JPY | 16.2 |
| Living with ≥ 6 | 0.8 |  | ≥ 8,000,000JPY; < 10,000,000JPY | 10.0 |
| Marriage (%) |  |  | ≥ 10,000,000JPY | 10.6 |
| Married | 46.2 |  | I don' know | 15.0 |
| Not Married | 53.8 |  | Number of Friends (%) |  |
| Education (%) |  |  | None | 12.4 |
| Junior high school graduation [item 1] | 1.6 |  | 1–5 persons | 44.2 |
| High school graduation [item 2] | 28.4 |  | 6–10 persons | 23.8 |
| Vocational school, junior college, and technical college graduation [item 3 and 4] | 22.4 |  | 11–20 persons | 9.0 |
| University graduation [item 5] | 42.0 |  | 21–30 persons | 3.4 |
| Postgraduate degree [item 6] | 5.0 |  | > 31 persons | 7.2 |
| Other [item7] | 0.6 |  |  |  |

Table 2. Descriptive statistics, factor loadings, reliability coefficients, and goodness of fit indices for the J-MVS-18 and J-MVS-15 (N = 500)

|  |  |  |  |  |  |  |  | Factor loadings for the J-MVS-18 | | |  | Factor loadings for the J-MVS-15 | | |
| --- | --- | --- | --- | --- | --- | --- | --- | --- | --- | --- | --- | --- | --- | --- |
| Item Number | Domain | Item | *Mean* | *SD* | *Skewness* | *Kurtosis (difference from 3.00)* |  | Success | Centrality | Happiness |  | Success | Centrality | Happiness |
| 1 | S | I admire people who own expensive homes, cars, and clothes. (3, 6, 9, 15) | 2.42 | 1.22 | 0.38 | -0.99 |  | 0.76 |  |  |  | 0.76 |  |  |
| 2 | S | Some of the most important achievements in life include acquiring material possessions. (15) | 3.11 | 1.07 | -0.25 | -0.58 |  | 0.66 |  |  |  | 0.70 |  |  |
| 3 | S | I don’t place much emphasis on the amount of material objects people own as a sign of success. (15) R | 2.77 | 1.03 | 0.07 | -0.60 |  | 0.52 |  |  |  | 0.47 |  |  |
| 4 | S | The things I own say a lot about how well I’m doing in life. (6, 9, 15) | 2.69 | 0.97 | 0.01 | -0.46 |  | 0.38 |  |  |  | 0.40 |  |  |
| 5 | S | I like to own things that impress people. (9, 15) | 2.40 | 1.10 | 0.42 | -0.62 |  | 0.48 |  |  |  | 0.47 |  |  |
| 6 | S | I don’t pay much attention to the material objects other people own. R | 2.61 | 1.03 | 0.24 | -0.53 |  | 0.54 |  |  |  |  |  |  |
| 7 | C | I usually buy only the things I need. R | 2.17 | 0.94 | 0.69 | 0.23 |  |  | 0.38 |  |  |  |  |  |
| 8 | C | I try to keep my life simple, as far as possessions are concerned. (9, 15) R | 2.52 | 0.98 | 0.41 | -0.12 |  |  | 0.35 |  |  |  | 0.23 |  |
| 9 | C | The things I own aren't all that important to me. (15) R | 3.42 | 0.93 | -0.16 | -0.20 |  |  | 0.22 |  |  |  | 0.21 |  |
| 10 | C | I enjoy spending money on things that aren’t practical. | 2.66 | 1.09 | 0.12 | -0.78 |  |  | 0.31 |  |  |  |  |  |
| 11 | C | Buying things gives me a lot of pleasure. (6, 9, 15) | 3.18 | 1.02 | -0.16 | -0.46 |  |  | 0.53 |  |  |  | 0.54 |  |
| 12 | C | I like a lot of luxury in my life. (3, 6, 9, 15) | 2.45 | 1.09 | 0.37 | -0.61 |  |  | 0.70 |  |  |  | 0.70 |  |
| 13 | C | I put less emphasis on material things than most people I know. (15) R | 3.00 | 0.88 | 0.10 | 0.16 |  |  | 0.40 |  |  |  | 0.35 |  |
| 14 | H | I have all the things I really need to enjoy life. (15) R | 3.55 | 1.01 | -0.13 | -0.64 |  |  |  | 0.35 |  |  |  | 0.34 |
| 15 | H | My life would be better if I owned certain things I don’t have. (6, 9, 15) | 3.41 | 0.99 | -0.14 | -0.35 |  |  |  | 0.76 |  |  |  | 0.76 |
| 16 | H | I wouldn’t be any happier if I owned nicer things. (15) R | 3.28 | 0.97 | -0.06 | -0.19 |  |  |  | 0.57 |  |  |  | 0.57 |
| 17 | H | I’d be happier if I could afford to buy more things. (3, 6, 9, 15) | 3.27 | 1.08 | -0.18 | -0.60 |  |  |  | 0.86 |  |  |  | 0.86 |
| 18 | H | It sometimes bothers me quite a bit that I can’t afford to buy all the things I’d like. (9, 15) | 2.86 | 1.20 | 0.15 | -0.90 |  |  |  | 0.66 |  |  |  | 0.66 |
|  |  |  |  |  | Phi matrix | Centrality |  | 0.79 |  |  |  | 0.82 |  |  |
|  |  |  |  |  |  | Happiness |  | 0.56 | 0.49 |  |  | 0.59 | 0.57 |  |
|  |  |  |  |  | Reliability coefficient | *Cronbach’s alpha* |  | 0.73 | 0.63 | 0.77 |  | 0.70 | 0.55 | 0.77 |
|  |  |  |  |  |  | *CR* |  | 0.74 | 0.60 | 0.79 |  | 0.71 | 0.52 | 0.79 |
|  |  |  |  |  |  | *AVE* |  | 0.34 | 0.20 | 0.45 |  | 0.36 | 0.22 | 0.45 |
|  |  |  |  |  | Goodness-of-fit indices | |  | df = 132, Chi-sq = 806.131 | | |  | df = 87, Chi-sq = 454.97 | | |
|  |  |  |  |  |  |  |  | AGFI = .775, CFI = .724, TLI = .680 | | |  | AGFI = .834, CFI = .810, TLI = .770 | | |
|  |  |  |  |  |  |  |  | RMSEA = .101, SRMR = .089 | | |  | RMSEA = .092, SRMR = .078 | | |

R: Reversed items. Numbers in parentheses indicate items adopted in various abbreviated versions of the original version (e.g., “3” means the three-item version of MVS) [9].

Table 3a. Comparisons of factor solutions by confirmatory factor analysis for the J-MVS in 6 models (N = 500)

| Model Number | | 1 | | | 2 | | | 3 | | | 4 | | | 5 (J-MVS-A6) | | 6 | | |
| --- | --- | --- | --- | --- | --- | --- | --- | --- | --- | --- | --- | --- | --- | --- | --- | --- | --- | --- |
| Cutoff criteria for factor loadings | | at least \|.3\| | | | at least \|.3\| | | | at least \|.4\| | | | at least \|.4\| | | | at least \|.5\| | | at least \|.5\| | | |
| Cutoff criteria for communalities | | - | | | at least .2 | | | - | | | at least .25 | | | - | | at least .3 | | |
| Number of Items | | 15 | | | 14 | | | 13 | | | 13 | | | 6 | | 6 | | |
| Extracted factors | | Success | Centrality | Happiness | Success | Centrality | Happiness | Success | Centrality | Happiness | Success | Centrality | Happiness | Success/Centrality | Happiness | Success |  | Happiness |
| Items | | 1, 2, 4, 5, 11, 12 | 6, 7, 8, 13 | 14, 15, 16, 17, 18 | 1, 2, 4, 5, 11, 12, 6 | 7, 8 | 14, 15, 16, 17, 18 | 1, 2, 4, 5, 11, 12 | 7, 8 | 14, 15, 16, 17, 18 | 1, 2, 4, 5, 11, 12 | 7, 8 | 14, 15, 16, 17, 18 | 1, 12 | 15, 16, 17, 18 | 3, 6 |  | 15, 16, 17, 18 |
| Goodness-of-fit indices | *df* | 87 | | | 74 | | | 62 | | | 62 | | | 8 | | 8 | | |
|  | *Chi-square* | 439.402 | | | 324.379 | | | 259.634 | | | 259.634 | | | 32.804 | | 39.496 | | |
|  | *Chi-square/df* | 5.051 | | | 4.384 | | | 4.188 | | | 4.188 | | | 4.101 | | 4.937 | | |
|  | *GFI* | 0.893 | | | 0.915 | | | 0.925 | | | 0.925 | | | 0.977 | | 0.976 | | |
|  | *AGFI* | 0.853 | | | 0.880 | | | 0.891 | | | 0.891 | | | 0.940 | | 0.937 | | |
|  | *CFI* | 0.829 | | | 0.870 | | | 0.889 | | | 0.889 | | | 0.973 | | 0.963 | | |
|  | *TLI* | 0.794 | | | 0.841 | | | 0.861 | | | 0.861 | | | 0.950 | | 0.931 | | |
|  | *SRMR* | 0.078 | | | 0.072 | | | 0.070 | | | 0.070 | | | 0.041 | | 0.043 | | |
|  | *RMSEA* | 0.090 | | | 0.082 | | | 0.080 | | | 0.080 | | | 0.079 | | 0.089 | | |
|  | *BIC* | 20233.604 | | | 18965.298 | | | 17599.140 | | | 17599.140 | | | 8189.156 | | 8049.681 | | |
| Reliability coefficients | Overall α | 0.809 | | | 0.803 | | | 0.791 | | | 0.791 | | | 0.787 | | 0.756 | | |
|  | α | 0.757 | 0.649 | 0.773 | 0.769 | 0.674 | 0.773 | 0.757 | 0.674 | 0.773 | 0.757 | 0.674 | 0.773 | 0.694 | 0.793 | 0.653 |  | 0.793 |
|  | AVE | 0.369 | 0.324 | 0.446 | 0.349 | 0.633 | 0.446 | 0.368 | 0.735 | 0.446 | 0.368 | 0.735 | 0.446 | 0.538 | 0.518 | 0.544 |  | 0.517 |
|  | CR | 0.765 | 0.652 | 0.786 | 0.776 | 0.757 | 0.786 | 0.764 | 0.824 | 0.786 | 0.764 | 0.824 | 0.786 | 0.698 | 0.805 | 0.693 |  | 0.806 |

Table 3b. Comparisons of factor solutions by confirmatory factor analysis for the J-MVS in 6 models with only positive-worded items (N = 500)

| Model Number | | 7 | | 8 | | 9 | | 10 | | | 11 (J-MVS-P6) | | 12 | | |
| --- | --- | --- | --- | --- | --- | --- | --- | --- | --- | --- | --- | --- | --- | --- | --- |
| Cutoff criteria for factor loadings | | at least \|.3\| | | at least \|.3\| | | at least \|.4\| | | at least \|.4\| | | | at least \|.5\| | | at least \|.5\| | | |
| Cutoff criteria for communalities | | - | | at least .2 | | - | | at least .25 | | | - | | at least .3 | | |
| Number of Items | | 10 | | 9 | | 9 | | 3 | | | 6 | | 3 | | |
| Extracted factors | | Success/Centrality | Happiness | Success/Centrality | Happiness | Success/Centrality | Happiness |  |  | Happiness | Success/Centrality | Happiness |  |  | Happiness |
| Items | | 1, 2, 4, 5, 10, 11, 12 | 15, 17, 18 | 1, 2, 4, 5, 11, 12 | 15, 17, 18 | 1, 2, 4, 5, 11, 12 | 15, 17, 18 |  |  | 15, 17, 18 | 1, 5, 12 | 15, 17, 18 |  |  | 15, 17, 18 |
| Goodness-of-fit indices | *df* | 34 | | 74 | | 26 | | 0 | | | 8 | | 0 | | |
|  | *Chi-square* | 158.393 | | 324.379 | | 123.596 | | 0.000 | | | 20.115 | | 0.000 | | |
|  | *Chi-square/df* | 4.659 | | 4.384 | | 4.754 | | - | | | 2.514 | | - | | |
|  | *GFI* | 0.941 | | 0.915 | | 0.950 | | 1.000 | | | 0.986 | | 1.000 | | |
|  | *AGFI* | 0.904 | | 0.880 | | 0.914 | | 1.000 | | | 0.964 | | 1.000 | | |
|  | *CFI* | 0.909 | | 0.870 | | 0.926 | | 1.000 | | | 0.986 | | 1.000 | | |
|  | *TLI* | 0.879 | | 0.841 | | 0.897 | | 1.000 | | | 0.974 | | 1.000 | | |
|  | *SRMR* | 0.058 | | 0.072 | | 0.054 | | 0.000 | | | 0.035 | | 0.000 | | |
|  | *RMSEA* | 0.086 | | 0.082 | | 0.087 | | 0.000 | | | 0.055 | | 0.000 | | |
|  | *BIC* | 13827.702 | | 18965.298 | | 12338.771 | | 4053.341 | | | 8360.946 | | 4053.341 | | |
| Reliability coefficient | Overall α | 0.801 | | 0.813 | | 0.813 | | 0.791 | | | 0.776 | | 0.791 | | |
|  | α | 0.743 | 0.791 | 0.757 | 0.791 | 0.757 | 0.791 |  |  | 0.791 | 0.687 | 0.791 |  |  | 0.791 |
|  | AVE | 0.324 | 0.575 | 0.368 | 0.575 | 0.368 | 0.575 |  |  | 0.573 | 0.448 | 0.575 |  |  | 0.573 |
|  | CR | 0.749 | 0.800 | 0.764 | 0.800 | 0.764 | 0.800 |  |  | 0.798 | 0.700 | 0.800 |  |  | 0.798 |

Table 5. Correlation between the J-MVS-A6 and J-MVS-P6 and personality (N = 500)

|  |  |  | Age | | Social comparison orientation (INCOM) | | | |  | Big Five personality (TIPI-J) | | | | | | | | | |
| --- | --- | --- | --- | --- | --- | --- | --- | --- | --- | --- | --- | --- | --- | --- | --- | --- | --- | --- | --- |
|  |  |  |  |  | Ability | | Opinion | |  | Openness | | Conscientiousness | | Extraversion | | Agreeableness | | Neuroticism | |
|  |  | Mean | 44.83 | | 2.71 | | 2.96 | |  | 7.47 | | 7.73 | | 7.10 | | 9.52 | | 8.65 | |
|  |  | SD | 14.11 | | 0.85 | | 0.93 | |  | 2.42 | | 2.53 | | 2.72 | | 2.20 | | 2.41 | |
|  |  | α | - | | 0.89 | | 0.79 | |  | 0.44 | | 0.56 | | 0.60 | | 0.34 | | 0.48 | |
| J-MVS-A6 | Overall | Pearson’s r | -0.29 | *** | 0.46 | *** | 0.15 | ** |  | 0.05 |  | -0.09 |  | -0.07 |  | -0.17 | ** | 0.26 | *** |
|  | Success/  Centrality |  | -0.19 | *** | 0.44 | *** | 0.23 | *** |  | 0.09 |  | 0.01 |  | 0.03 |  | -0.12 |  | 0.15 | * |
|  | Happiness |  | -0.29 | *** | 0.36 | *** | 0.06 |  |  | 0.01 |  | -0.12 |  | -0.11 |  | -0.16 | ** | 0.26 | *** |
| J-MVS-P6 | Overall | Pearson’s r | -0.28 | *** | 0.51 | *** | 0.21 | *** |  | 0.10 |  | -0.07 |  | -0.03 |  | -0.15 | * | 0.25 | *** |
|  | Success/  Centrality |  | -0.19 | *** | 0.49 | *** | 0.29 | *** |  | 0.17 | ** | 0.03 |  | 0.07 |  | -0.10 |  | 0.14 | * |
|  | Happiness |  | -0.29 | *** | 0.37 | *** | 0.07 |  |  | 0.00 |  | -0.14 | * | -0.13 |  | -0.15 | * | 0.28 | *** |

¶Evaluation by adjusted p-value based on Holm method: ***: p < .001, **: p < .01, *: p<.05

Table 7. Results of measurement invariance tests (N = 500)

|  |  | Model Fit Measures | | | | | |  | Model Differences | | | | |
| --- | --- | --- | --- | --- | --- | --- | --- | --- | --- | --- | --- | --- | --- |
|  |  | χ2 | *df* | *p* | χ2/*df* | CFI | RMSEA |  | Δχ2 | Δ*df* | *p* | ΔCFI | ΔRMSEA |
| Sex | Configural invariance | 28.005 | 16 | 0.032 | 1.750 | 0.986 | 0.039 |  |  |  |  |  |  |
|  | Full metric invariance | 48.987 | 22 | 0.001 | 2.227 | 0.969 | 0.050 |  | 20.982 | 6 | 0.002 | -0.017 | 0.011 |
|  | Partial metric invariance *1 | 32.745 | 20 | 0.036 | 1.637 | 0.986 | 0.036 |  | 4.740 | 4 | 0.315 | 0.000 | -0.003 |
|  | Partial scalar invariance *1 | 38.557 | 24 | 0.030 | 1.607 | 0.983 | 0.035 |  | 5.812 | 4 | 0.214 | -0.003 | -0.001 |
| Age | Configural invariance | 59.961 | 40 | 0.022 | 1.499 | 0.975 | 0.032 |  |  |  |  |  |  |
|  | Full metric invariance | 102.487 | 64 | 0.002 | 1.601 | 0.952 | 0.035 |  | 42.525 | 24 | 0.011 | -0.023 | 0.003 |
|  | Partial metric invariance *2 | 81.325 | 60 | 0.035 | 1.355 | 0.974 | 0.027 |  | 21.364 | 20 | 0.376 | -0.001 | -0.005 |
|  | Partial scalar invariance *2 | 137.93 | 80 | 0.000 | 1.724 | 0.928 | 0.038 |  | 56.604 | 20 | 0.000 | -0.046 | 0.011 |

*1: Released loadings for items 1 and 17. *2: Released loadings for item 5.
