# Supplementary material for: The Japanese version of the Material Values Scale: construct assessment and relationship with age, personality, and subjective well-being
Source: BMC Psychol. 2022 Aug 13;10:200. doi: 10.1186/s40359-022-00889-3 (PMC9375416; doi:10.1186/s40359-022-00889-3)
Supplement: Supplementary file 2 — Additional file 2. The Japanese version of the Material Value Scale (J-MVS). This file contains all MVS items translated into Japanese. [file 40359_2022_889_MOESM2_ESM.docx]

**Supplementary Information**

The Japanese version of the Material Value Scale (J-MVS)

Items:

1) *高価な家，車，衣服を持つ人々に私は憧れを抱く [I admire people who own expensive homes, cars, and clothes.]

2) 人生における最も重要な業績の中には，財産を獲得していくことが含まれる [Some of the most important achievements in life include acquiring material possessions.]

3) [R]私は，人々が持っている物質的な物の量を成功の証として考えることはあまりない。 [I don’t place much emphasis on the amount of material objects people own as a sign of success.]

4) 私が持っているものは，私が人生でどれだけうまくやっているかを表している [The things I own say a lot about how well I’m doing in life.]

5) *私は，人々に印象づけるようなものを持つのが好きだ [I like to own things that impress people.]

6) [R]私は，他の人々が持っている物質的なものに，それ程注意を払っていない [I don’t pay much attention to the material objects other people own.]

7) [R]私は普段，自分が必要としているものだけを買う [I usually buy only the things I need.]

8) [R]所有物に関する限り，私は自分の人生をシンプルに保とうとしている [I try to keep my life simple, as far as possessions are concerned.]

9) [R]私が所有しているものは，私にとってそれ程重要なものではない [The things I own aren’t all that important to me.]

10) 私は，実用的でないものにお金をつかって楽しむ [I enjoy spending money on things that aren’t practical.]

11) ものを買うことは，私に大きな喜びをもたらす [Buying things gives me a lot of pleasure.]

12) *私はとても贅沢な生活が好きだ [I like a lot of luxury in my life.]

13) [R]私は，私の知る殆どの人々よりも，物質的なものに価値を置いていない [I put less emphasis on material things than most people I know.]

14) [R]私は，人生を楽しむために本当に必要とするものをすべて持っている [I have all the things I really need to enjoy life.]

15) *もし私が今持っていないものを持てるようになったら，私の人生はもっと良くなるだろう [My life would be better if I owned certain things I don’t have.]

16) [R]より良いものを持てたとしても，私は今より幸せになることはない [I wouldn’t be any happier if I owned nicer things.]

17) *もし，もっと多くのものを買う余裕があったとしたら，私はもっと幸せになれるだろう [I’d be happier if I could afford to buy more things.]

18) *欲しいもの全てを買う余裕がないことが，時々私をかなり悩ませる [It sometimes bothers me quite a bit that I can’t afford to buy all the things I’d like.]

Note. Original items are presented in brackets. Items are rated on a 5-point scale. Items with [R] are reverse-coded. *: The items adopted in J-MVS-P6
